#  QSAR Study on Anti-HIV-1 Activity of 4-Oxo-1,4-dihydroquinoline and 4-Oxo-4*H*-pyrido[1,2-*a*]pyrimidine Derivatives Using SW-MLR, Artificial Neural Network and Filtering Methods 

**Published:** 2015

**Authors:** Zahra Hajimahdi, Amin Ranjbar, Amir Abolfazl Suratgar, Afshin Zarghi

**Affiliations:** a *Department of Medicinal Chemistry, School of Pharmacy, Shahid Beheshti University of Medical Sciences, Tehran, Iran. *; b *Electrical Engineering Department, Amirkabir University of Technology, Tehran, Iran. *; c *The Center of Excellence on Control and Robotics, Electrical Engineering Department, Amirkabir University of Technology, Tehran, Iran.*

**Keywords:** QSAR, 4-Oxo-1, 4-dihydroquinoline, Oxo-4*H*-pyrido[1, 2-*a*]pyrimidine, Neural network

## Abstract

Predictive quantitative structure–activity relationship was performed on the novel4-oxo-1,4-dihydroquinoline and 4-oxo-4*H*-pyrido[1,2-*a*]pyrimidine derivatives to explore relationship between the structure of synthesized compounds and their anti-HIV-1 activities. In this way, the suitable set of the molecular descriptors was calculated and the important descriptors using the variable selections of the stepwise technique were selected. Multiple linear regression (MLR) and artificial neural network (ANN) as nonlinear system were used for constructing QSAR models. The predictive quality of the quantitative structure–activity relationship models was tested for an external set of five compounds, randomly chosen out of 25 compounds. The findings exhibited that stepwise-ANN model was more efficient at prediction activity of both training and test sets with high statistical qualities. Based on QSAR models results, electronegativity, the atomic masses, the atomic van der Waals volumes, the molecular symmetry and polarizability were found to be important factors controlling the anti-HIV-1 activity.

## Introduction

The causative agent of acquired immune deficiency syndrome (AIDS) is the human immunodeficiency virus type 1 (HIV-1)(1-3). During the past 3 decades, the combination of antiretroviral drugs in HAART (highly active antiretroviral therapy) regimens has transformed the management of HIV infection from a fatal disease to a manageable chronic condition(4,5). However, resistance to marketed anti-HIV drugs is increasing at an alarming rate. Thus, there is a need to develop new agents possessing modified scaffolds which work by different mechanisms. 

Although there are several *in-vivo* and *in-vitro* assay methods available for screening the biological activity of chemicals, they are costly and time-consuming. Therefore, computational methods have been developed as an alternative tool for predicting properties of chemicals (6). One of these computational methods is Quantitative structure–activity relationship (QSAR) study which plays a critical role in the rational drug design (7-8). The major aim of QSAR study is to develop quantitative models to predict compounds' properties such as biological activity, and these models can contribute to reduction of the time of drug discovery(9-10). Biological activities are a function of molecular descriptors which derived from the chemical structure of a set of molecules. Therefore, a model containing those calculated descriptors can beused to estimate activities of new compounds. The application of QSAR models usually requires variable selection for constructing coherent models (13-15). Through the years Different calculated descriptors and variable selection methods were used to build QSAR models capable of accurate prediction of anti-HIV-1 activity of compounds (12-13). In this study, we employed the stepwise (SW) selection method for the variable selection in the multiple linear regression (MLR) method. In addition, wavelet transformation and artificial neural network was used as nonlinear system for QSAR modeling. The aim of this paper is to search for an efficient method to build an accurate quantitative relationship between the molecular structure and the anti-HIV-1 activity of novel 4-oxo-1,4-dihydroquinoline and 4-oxo-4*H*-pyrido[1,2-*a*]pyrimidine derivatives. The findings can be helpful for designing new active derivatives.

## Experimental

*Data set*

A data set of twenty five 4-oxo-1,4-dihydroquinoline and 4-oxo-4*H*-pyrido[1,2-*a*]pyrimidine derivatives which have been synthesized and evaluated as anti-HIV-1 agents in our laboratory were selected (16-17). Anti-HIV-1 activities of the molecules as inhibition rate of p24 expression (IR) in cell culture were converted into corresponding log inhibition rate of p24 expression (log IR). The total set of molecules was randomly divided into a training set (20 compounds) for generating QSAR model and a test set (5 compounds) for validating the quality of the model. The general chemical structures and biological activity values of all of the compounds are shown in Table 1.

*Software*

The geometry optimization was performed with HYPERCHEM (version 8.0; Hyper Chem, Alberta, Canada)(18). For the calculation of the molecular descriptors, the DRAGON 2.1 software was used(19). The SPSS software (version 13.0; SPSS Inc., Chicago, IL, USA) was employed for the simple MLR analysis(20). The SW-ANN calculations were performed in the MATLAB (version 7.0, Math Works, Natick, MA, USA).

*Molecular descriptors and geometry optimization*

The chemical structures of the molecules were drawn using the Hyper chem 8.0 software. The pre-optimization was conducted using the molecular mechanics force field (MM+)procedure included in Hyper chem, and then the molecular structures were finally optimized by the semi-empirical method AM1 using the Polak-Ribiere algorithm until the root mean square gradient was 0.01 kcal mol^-1^. The resultant geometry was transferred into the Dragon software package to calculate the descriptors in constitutional descriptors, topological descriptors, molecular walk counts, BCUT descriptors, Galves topological charge indices, 2D autocorrelations, charge descriptors, aromaticity indices, Randic molecular profiles, geometrical descriptors, 3D-MoRSE descriptors,WHIM descriptors, GETAWAY descriptors, empirical descriptors. The 842 descriptors were first analyzed for the existence of constant or near constant variables. Secondly, correlation among descriptors and with the activity of the molecules was calculated and collinear descriptors (*i.e*. correlation coefficient between descriptors is greater than 0.9) were detected. Descriptors that contain a high percentage (>90%) of identical values for all the 25 molecules were discarded. Among the collinear descriptors, the one exhibiting the highest correlation with the activity was remained and others were removed from the data matrix. Then, the remaining descriptors were collected in an n×m data matrix (D), where n = 25 and m = 243 are the numbers of the compounds and the descriptors, respectively.

*Artificial neural networks as a nonlinear system predictor*

**Figure 1:**
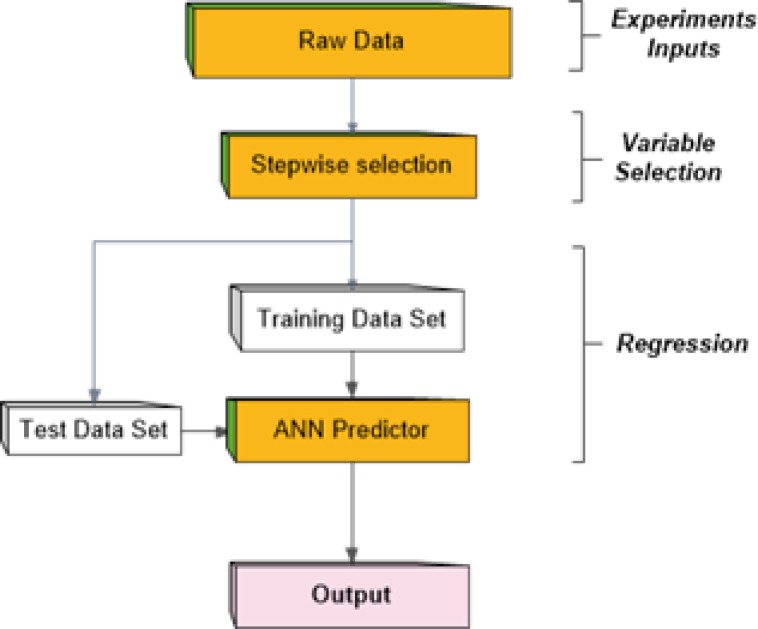
Procedure of the method

In mathematics, regression analysis is a process for estimating the relationships among variables. That is, regression analysis can be used to infer causal relationships between the independent and dependent variables. Regression analysis is widely used for prediction and forecasting. It includes many techniques for creating an appropriate model and analyzing several independent variables. Among these techniques, artificial neural networks (ANN) are frequently applied to carry out this problem due to function approximation capability. In this paper we considered a multi-layer perceptron network (MLP) which is the most well-known form of ANNs as a predictor to generate future values of the output (21-23). On the other hands, it seems that existence of large number of descriptors as the inputs to predictor network brings more complexity in MLP network. To avoid this, a stepwise variable selection stage is used to choose most proper descriptors as network inputs. The method proceeds as depicted in Figure 1 schematically.

## Results

*Variable selection*

The step-by-step iterative building of a regression model that involves automated choice of independent variables. Based on statistical significance of each variable in a regression, stepwise method can be achieved either by trying out one independent variable at a time and including it in the regression model or removing it based on the obtained value for parameter of p. In this systematic manner, the p-value of an F-statistic is computed to test models with the present variables. The available data set is a matrix with size of 20×243 where 20 and 243 are total number of training group and variables respectively. At the end of this stage, the best set of the calculated descriptors was selected in the dataset as network inputs in order to train MLP network.

*Results of SW-MLR method*

The MLR analysis with a stepwise selection and the variables elimination was employed to relate the anti-HIV-1 activity to a different set of descriptors. The SW-MLR analysis led to the derivation of one model, with four variables (the closest to the ratio of five training molecules for each descriptor) and good statistical parameters for the training set and with low generalization and prediction ability for the prediction set (Tables 1 and 2). It is described by the following equation:

Log Inhibition Rate = –6.21(±2.53) + 0.92(±0.14) GATS6v – 25.12(±6.36) JGI5 + 8.10(±2.61)ISH – 41.21(±3.20)R6p+.

**Table 1:** Chemical structures and experimental and predicted activities for 4-oxo-1,4-dihydroquinoline and 4-oxo-4H-pyrido[1,2-a]pyrimidine analogs by SW-MLR.

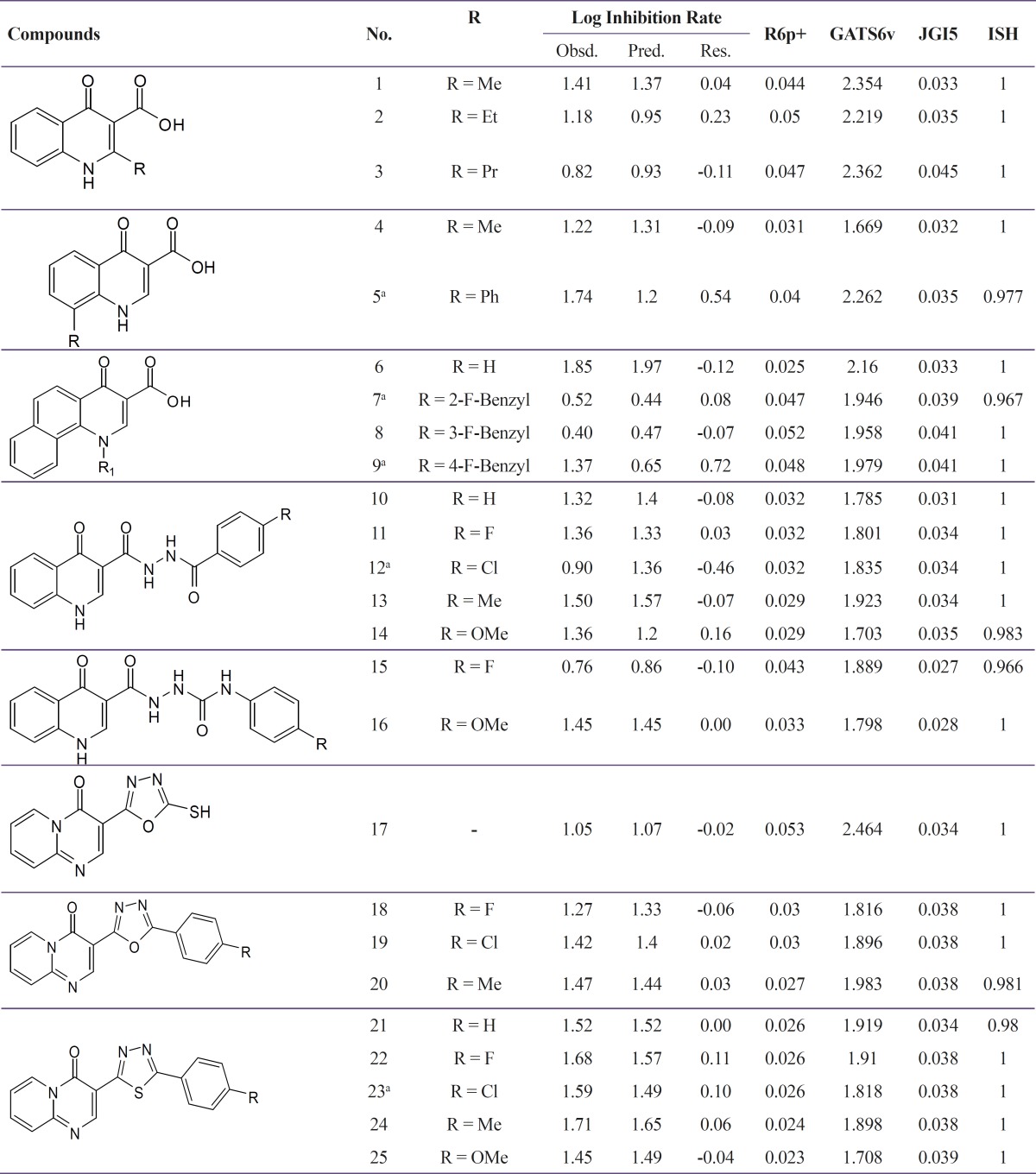

a The prediction set (test set)

**Table 2:** Statistical parameters of SW-MLR model

**Training set**		**Test set**	**F**	**Q** ^2^_LOO_
SEE	R^2^	R^2^		
0.10	0.93	0.30	47.44	0.84

**Figure 2:**
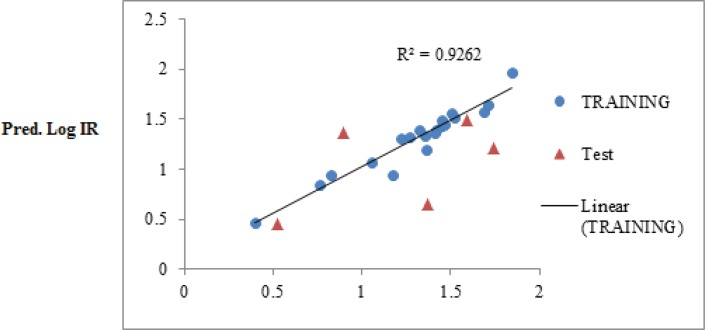
The predicted Log IR values by the SW-MLR modeling versus the observed Log IR values

The obtained statistical parameter of the leave-one-out cross-validation test (Q^2^) on SW-MLR model was 0.84, which indicates reliability of the proposed model. The plots of the predicted log IR versus the experimental log IR, obtained by the SW-MLR modeling, are demonstrated in Figure 2.

However, this procedure produced good results for the training set, but it did not display good results for the prediction set. Therefore, the SW-MLP was used to build an appropriate model for both training and test set.

*Results of SW-MLP method*

**Figure 3:**
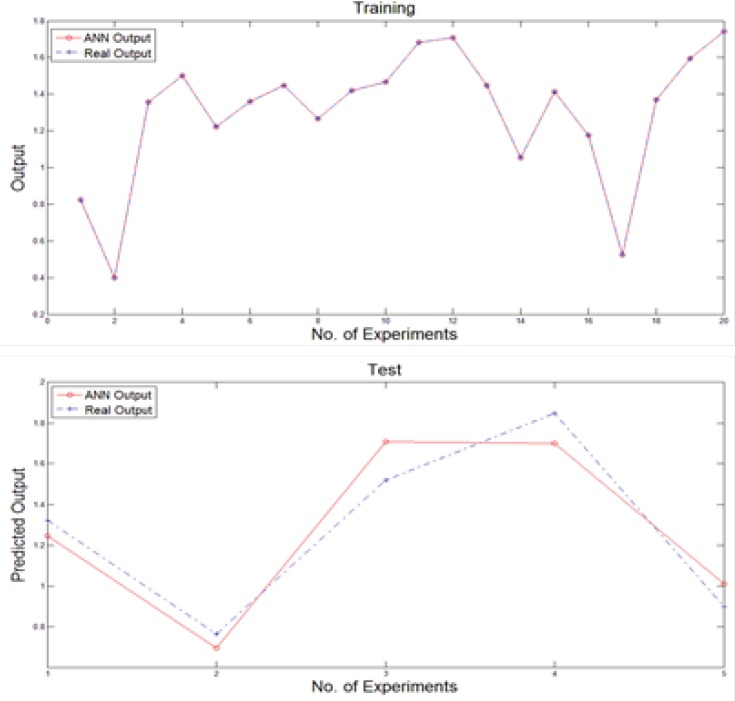
Training and test obtained results

We used a multi-layer perceptron neural network with back-propagation training algorithm to predict new output values after training stage. Generally, the input vector to the network was provided by the nominated variables of previous stage while the output layer was composed of a single neuron. The number of hidden layers and the number of nodes in each hidden layer affect the generalization capability of the network. For this purpose, the structure of the net is considered as 40×30×5×1 neurons. Moreover, the training of the MLP network involves finding values of the connection weights, which minimize an error function between the actual network output and the corresponding target value in the training set. Hence, the training algorithm used is based on resilient propagation algorithm. To generating neural network, the data set was broken into two sections as the number of 20 experiments were used to train and rest of them were applied to test the network performance. Figure 3 illustrates the obtained results of feeding training and test inputs to MLP network during these stages. 

## Discussion

QSAR results can provide useful chemical visions for designing new compounds. For this purpose, interpretation of the descriptors appeared in the resulting models was discussed below(24).

GATS6v (Geary autocorrelation of lag 6 weighted by van der Waals volume) is one of the 2D autocorrelation descriptors which has appeared in the SW-MLR model. In this descriptor, the Geary coefficient is a distance-type function, that function is any physico-chemical property calculated for each atom of the molecule, such as atomic mass, polarizability, etc. Therefore, the molecule atoms represent the set of discrete points in space and the atomic property the function evaluated at those points. The physico-chemical property in this case is van der Waals volume.GATS6v displays a positive sign, which indicates that the increasing the van der Waals volume value causing an improvement in log IR value.

JGI5 (mean topological charge index of order 5) is the second descriptor in the SW-MLR model. Topological charge indices were proposed to evaluate the charge transfer between pairs of atoms, and therefore the global charge transfer in the molecule. The JGI5 is closely related to the molecular branching. This descriptor has a significant negative effect on the anti-HIV-1 activity of analogs. The negative sign suggests that the Log IR value is inversely related to this descriptor. Subsequently, molecular branching results in log IR value decrease.

The third descriptor is ISH (standardized information content on the leverage equality) which has a positive influence on analogs activities. Standardized information content on the leverage equality mainly encode information on molecular symmetry; if all the atoms have different leverage values, *i.e*., the molecule does not have any element of symmetry, ISH is equal to one. Otherwise, if all the atoms have equal leverage values (a perfectly symmetric theoretical case), ISH is equal to zero. Based on the SW-MLR model decreasing molecular symmetry improves anti-HIV-1 activity of compounds.

The forth descriptor of the SW-MLR model was R6p+ (R maximal autocorrelation of lag 6/weighted by polarizability). This descriptor is a GETAWAY type and is related to the polarizability of the atoms in the molecule.This descriptor displays a main negative sign, which indicates that the log IR is inversely related to the polarizability of the molecules.

It is concluded that based on SW-MLR model polarizability, the molecular symmetry and branching and the atomic van der Waals volumes have effects on the anti-HIV-1 activity of the studied compounds. The results of SW-MLP model confirmed that polarizability, the molecular symmetry and the atomic van der Waals volumes are important molecular properties which influence anti-HIV-1 activity of training and test set compounds. SW-MLP model also indicated that atomic masses and electronegativity of atoms have significant effects on the activity of compounds.

## Conclusion

The QSAR analysis was performed on a series 4-oxo-1,4-dihydroquinoline and 4-oxo-4*H*-pyrido[1,2-*a*] pyrimidine derivatives with the usage of the MLR and artificial neural network and filtering methods. Over 842 theoretically derived descriptors were calculated for each molecule. The best set of the calculated descriptors was selected with the step-wise method. Multiple linear regression and artificial neural network as nonlinear system were used for QSAR modeling. Two models exhibited good statistical qualities for the training group. In parallel, the SW-MLP (nonlinear system) was found to be superior to the SW-MLR with reference to the test set predictions. Based on QSAR models results, electronegativity, the atomic masses, the atomic van der Waals volumes, the molecular symmetry and polarizability were found to be important factors controlling the anti-HIV-1 activity.
